# Augmenting colonoscopy with artificial intelligence and robotic assistance: a human-factors scoping review toward a task-based shared autonomy paradigm

**DOI:** 10.1007/s11701-026-03923-3

**Published:** 2026-10-12

**Authors:** Luigi Manfredi, Alfredo Annicchiarico, Gianpiero Gravante

**Affiliations:** 1 Division of Respiratory Medicine and Gastroenterology, Faculty of Health, University of Dundee, Dundee, UK; 2 Department of Surgery, Azienda Sanitaria Locale di Parma, Vaio Hospital, Fidenza, Italy; 3 Department of General Surgery, Azienda Sanitaria Locale ASL Lecce, Lecce, Italy

**Keywords:** Robotic colonoscopy, clinical adoption, human factors, procedural efficiency, cognitive workload, artificial intelligence

## Abstract

Colonoscopy is central to colorectal cancer prevention but remains a high-demand psychomotor and cognitive procedure, characterized by operator dependency, variable performance, and substantial physical and mental workload. Increasing evidence links procedural quality and safety to clinician-related factors, including musculoskeletal strain, fatigue, and non-technical skills. Concurrently, artificial intelligence (AI) and robotic technologies are emerging as potential tools to address these limitations. We conducted a scoping review with narrative synthesis of literature on human factors, ergonomics, AI, robotic technologies, and shared autonomy in colonoscopy and surgical robotics. Evidence synthesis: Colonoscopy performance remains limited by non-negligible miss rates and variability driven by both technical and human factors. Ergonomic constraints and cognitive workload contribute significantly to operator strain and may affect vigilance and decision-making. AI systems—including computer-aided detection (CADe), diagnosis (CADx), and quality monitoring—have demonstrated improvements in detection metrics and procedural standardisation, although concerns regarding cognitive offloading and potential deskilling persist. In parallel, emerging robotic and soft-robotic platforms aim to enhance navigation, improve procedural ergonomics, and reduce operator musculoskeletal strain, although clinical evidence remains limited. These technologies converge within a task-based shared-autonomy paradigm integrating physical, perceptual, and cognitive augmentation, with different procedural tasks operating at different levels of human–system control. A task-based shared-autonomy framework may provide a coherent model for integrating AI and robotics into colonoscopy while preserving human supervision and clinical judgement. Future developments should prioritise human-centred design, workflow integration, and multidimensional evaluation strategies to ensure safe and effective clinical implementation.

**Supplementary Information:**

The online version contains supplementary material available at https://doi.org/10.1007/s11701-026-03923-3.

## Introduction

Colorectal cancer remains a major global health challenge, ranking among the leading causes of cancer incidence and mortality worldwide [1], with a concerning rise in early-onset disease in several high-income countries [2]. Population screening represents a cornerstone of prevention and early detection strategies, and colonoscopy plays a central role within screening pathways due to its unique ability to combine diagnosis with immediate therapeutic intervention, effectively interrupting the adenoma–carcinoma sequence [3, 4].

Despite continuous technological evolution, colonoscopy remains a highly operator-dependent procedure, characterized by significant variability in performance [5], non-negligible miss rates [6], and procedure-related risks [7]. These limitations are not purely technological but are deeply rooted in the human factors that underpin procedural performance. Colonoscopy imposes substantial physical demands, including repetitive force application and non-ergonomic postures, alongside considerable cognitive workload, requiring sustained visual attention, spatial navigation, and real-time decision-making within a dynamic and often unpredictable anatomical environment [8–10]. In this context, recent advances in artificial intelligence (AI) [11] and robotic-assisted endoscopy [12] have emerged as potential strategies to mitigate these constraints. AI systems have demonstrated the ability to augment lesion detection and standardize aspects of procedural quality [13, 14], while robotic and soft-robotic platforms aim to reduce mechanical strain and improve navigation through alternative locomotion strategies [15].

Existing reviews have largely addressed AI, robotic colonoscopy, and autonomous endoscopy as separate technological domains [12, 16]. The concept of “*shared autonomy*” is well established in the broader human–robot interaction and surgical robotics literature [17] and has also been applied to robotic flexible endoscopy [18, 19], where control of specific tasks is distributed between the human operator and the robotic system [18, 19]. Building on this concept, the present scoping review uses *shared autonomy* as an integrative framework to examine how cognitive and physical procedural tasks may be distributed between clinicians, AI-based assistance, and robotic systems. Accordingly, colonoscopy is used here as an illustrative procedural domain in which task-based *shared autonomy* can be examined across physical, perceptual, and cognitive forms of augmentation, and where different procedural tasks may operate at different levels of *shared autonomy* while preserving overarching human supervision.

## Materials and methods

This study was conducted as a scoping review with narrative synthesis to map and integrate the heterogeneous literature on human factors, ergonomics, AI, robotic technologies, and *shared autonomy* in colonoscopy, while incorporating relevant concepts from the broader surgical robotics literature. A scoping approach was considered appropriate because of the multidisciplinary nature of the topic and the heterogeneity in study designs, technological maturity, and reported outcomes.

### Search strategy

A structured literature search was performed in PubMed/MEDLINE, Embase, and Scopus, from database inception to 15 August 2026. Controlled vocabulary terms, where available, and free-text keywords were used to identify studies addressing colonoscopy and gastrointestinal endoscopy in relation to human factors, cognitive workload, ergonomics, artificial intelligence, computer-aided detection and diagnosis, robotic endoscopy, autonomous and semi-autonomous systems, and human–robot interaction. Because concepts of shared and graded autonomy have largely developed within the surgical robotics literature, the search also included terms related to surgical robotics, levels of autonomy, task autonomy, shared autonomy, shared control, and human–robot collaboration. The two conceptual components were combined using Boolean operators and adapted to the syntax and indexing system of each database. Complete database-specific search strategies are reported in Supplementary Table S1.

#### Eligibility criteria and study selection

Publications were eligible when they contributed to one or more of six predefined thematic domains: (i) contemporary performance and limitations of colonoscopy; (ii) human factors and cognitive workload; (iii) ergonomics and physical workload; (iv) cognitive and perceptual augmentation through AI; (v) physical augmentation through robotic or soft-robotic technologies; and (vi) shared autonomy, human–AI–robot interaction, and relevant concepts derived from surgical robotics. Only publications in English were considered for the scoping review. Eligible evidence included clinical and observational studies, human-factors and ergonomic studies, systematic reviews and meta-analyses, guidelines and position statements, technical and engineering studies, and preclinical or proof-of-concept investigations. Editorials and perspective articles were included only when they provided relevant conceptual information on human–technology interaction or autonomy frameworks. Publications were excluded when they were unrelated to gastrointestinal endoscopy or did not provide concepts transferable to the proposed shared-autonomy framework.

After duplicate removal, AA and GG independently screened titles and abstracts, followed by full-text assessment of potentially eligible publications. Disagreements were resolved by discussion, with LM acting as a third reviewer when consensus could not be reached. Reference lists of key reviews, guidelines, position statements, and eligible articles were also screened to identify additional relevant publications, which underwent the same eligibility assessment. The complete study-selection process was summarized in a PRISMA-ScR flow diagram, reporting the number of records identified from each database, duplicates removed, records screened, full-text articles assessed for eligibility, full-text exclusions with reasons, and publications ultimately included in the review.

#### Data extraction and evidence synthesis

For each included publication, relevant data were extracted on study design, clinical or technological context, procedural task, type of augmentation, human and system roles, degree of autonomy, and maturity of the supporting evidence. Studies were mapped to one or more of the six predefined thematic domains; therefore, domain-specific counts could overlap. Given the heterogeneity of the evidence, findings were synthesized narratively and no quantitative pooling was planned. Evidence was interpreted according to its maturity, distinguishing established clinical evidence from early clinical feasibility studies and preclinical or engineering proof-of-concept investigations. A formal risk-of-bias assessment was not performed because the review aimed to map and conceptually integrate heterogeneous evidence rather than estimate a pooled treatment effect.

## Results

**Fig. 1:**
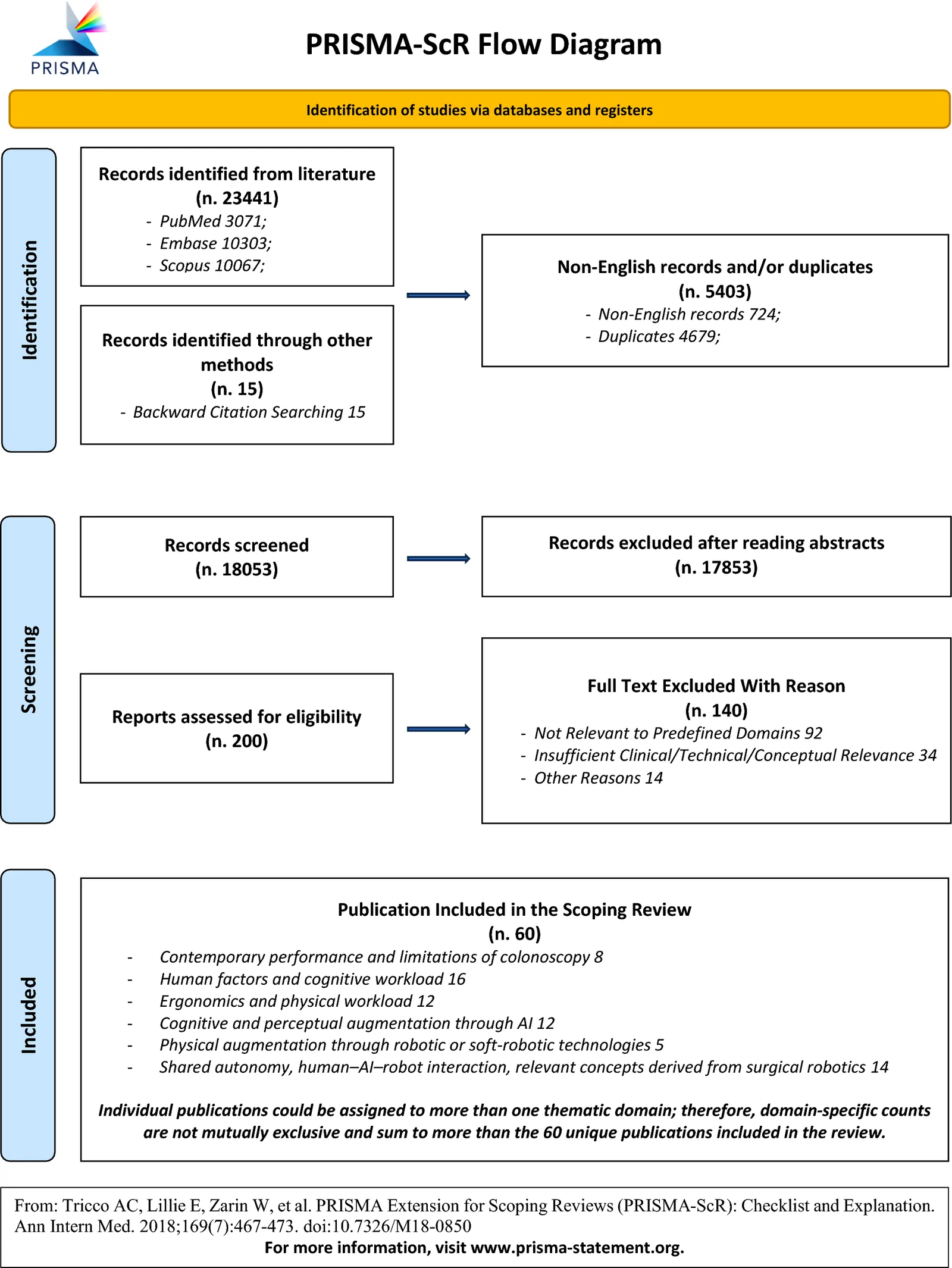
PRISMA-ScR flow diagram showing the identification, screening, eligibility assessment, and selection of publications included in the scoping review

A PRISMA flow diagram outlining the study selection process is shown in Fig. 1. The search identified 23,441 records, of which 18,053 remained after non-English language and duplicate removal. Following title/abstract and full-text screening, 60 publications were included in the review.

### Shared autonomy in the context of surgical robotics

The concept of *shared autonomy* in colonoscopy should be interpreted within the broader evolution of autonomy in surgical robotics [20–22]. Existing taxonomies describe a continuum ranging from direct human control or teleoperation to progressively higher levels of robot decision-making and task execution [20–23]. In commonly used surgical-robotics classifications, lower levels correspond to systems in which the robot assists or constrains surgeon-controlled motion, whereas intermediate levels allow the system to execute predefined subtasks under human supervision; higher levels imply conditional or ultimately full autonomy [20–23]. Contemporary clinical robotic systems remain predominantly within the lower levels of this spectrum, with the surgeon retaining responsibility for procedural planning, interpretation, and intervention [23].

This distinction is particularly relevant because autonomy in surgery is rarely an all-or-none property of an entire procedure [22, 23]. Rather, it is increasingly conceptualized at the level of individual tasks or subtasks [20–23]. In multi-port robotic surgery, for example, the surgeon remains in direct control of instrument manipulation while robotic systems provide motion scaling, tremor filtering, stable visualization, constrained kinematics, and platform-mediated coordination [22, 23]. In single-port and flexible robotic systems, the relationship becomes more complex because navigation, camera control, collision avoidance, instrument positioning, and workspace optimization may increasingly be supported by semi-autonomous functions [24]. Human–robot collaboration therefore extends beyond the simple distinction between manual and autonomous operation [25]. Shared control may involve cooperative motion, virtual fixtures [26], haptic or visual guidance, automated camera positioning, trajectory assistance, or conditional execution of specific subtasks while the clinician maintains supervisory authority [22, 25]. Accordingly, surgical-robotics autonomy taxonomies support assigning levels of autonomy to individual tasks rather than to the procedure as a whole and provide the conceptual basis for examining different levels of human–system control in colonoscopy [21–23].

#### Contemporary performance and limitations in colonoscopy

Despite major advances in imaging, accessories, and therapeutic endoscopy, the basic mechanical interface of the colonoscope remains largely unchanged and continues to require physically demanding push–torque manoeuvres and substantial operator skill, particularly in anatomically challenging cases [27, 28]. Colonoscopy also remains limited by non-trivial polyp and adenoma miss rates and procedure-related adverse events, with outcomes influenced by lesion characteristics, bowel preparation, inspection technique, patient factors, and therapeutic intervention [6, 29, 30]. Sedation and anaesthesia introduce additional monitoring and coordination requirements, further reinforcing the relevance of human-factor and system design considerations [31].

#### Physical workload in colonoscopy

*Physical workload* includes repetitive upper-limb motion, torque transmission, grip force, prolonged standing, and awkward postures driven by monitor/bed placement and colon looping mechanics [10]. Endoscopists frequently perform colonoscopy in postures characterized by sustained standing, neck rotation or flexion towards mispositioned monitors, shoulder abduction, wrist deviation, repetitive torquing, and pinch-grip force at the control head—often compounded by long lists and limited recovery time. These exposures plausibly explain why endoscopy-related musculoskeletal injury (ERI) is repeatedly reported as prevalent across settings and career stages [32]. A 2025 systematic review synthetising survey data across thousands of endoscopists reported a pooled career-long prevalence of ERI exceeding 60%, with pain syndromes predominating [33]. Cross-specialty reviews similarly identify high ERI rates across endoscopic domains and highlight modifiable environmental and behavioural risk factors [34]. Current ASGE guidance frames ergonomics as both a patient-safety and workforce-sustainability issue and supports formal ergonomics education, neutral posture, adjustable equipment, and other practical interventions despite the generally low certainty of the available evidence [10]. Training and simulation studies suggest that ergonomic behaviour can be improved, although equipment and workspace design remain necessary to address residual physical risk [35–37].

Comparable human-factor considerations apply to robotic surgery, although the ergonomic profile differs [38]. Robotic-assisted surgery may reduce several physical demands associated with conventional and laparoscopic surgery [39], while simultaneously introducing distinct cognitive and organizational ergonomic requirements [40]. Thus, technological assistance should not be assumed to eliminate ergonomic burden, but rather to redistribute it, reinforcing the importance of human-centred system design and ergonomic training across both endoscopic and robotic platforms [38]. This creates an important parallel between physical and cognitive augmentation: reducing one component of operator burden may generate another [25, 38]. Robotic assistance may decrease repetitive force application and awkward upper-limb movements, yet introduce prolonged static posture, visual concentration, interface dependence, and altered team interaction [38, 41]. Ergonomic benefit should therefore be evaluated at the level of the complete human–technology system rather than inferred from reduction of a single physical demand [38].

#### Physical augmentation: robotics

In contrast to AI-assisted detection, the evidence supporting robotic and semi-autonomous colonoscopy remains substantially less mature [12, 42]. Much of the literature consists of engineering development, preclinical validation, feasibility studies, and relatively small early clinical series; these findings should therefore be interpreted as evidence of technological capability and feasibility rather than established clinical effectiveness [12, 15, 42]. Robotics and embodied assistance address a different bottleneck: the mechanical difficulty and discomfort associated with advancing a flexible colonoscope through a tortuous, deformable colon. Reviews describe multiple robotic colonoscopy concepts—self-propelled “inchworm” devices, soft robotic locomotion, tethered magnetic actuation, and capsule robotics—aiming to reduce patient discomfort and looping while potentially reducing operator force and awkward movements [12]. Recent engineering work demonstrates semi-autonomous manipulation of electromagnetically actuated soft-tethered colonoscopes using visual servo control, explicitly targeting reduced user workload and higher autonomy levels [15]. Soft-robotic self-propelled platforms employing double-balloon locomotion have been investigated as an alternative to conventional push-based colonoscopy, with the potential to improve force distribution, reduce mucosal stress, and enhance safety while altering the procedural workflow [12, 43]. Early clinical experiences with novel robotic systems in difficult or previously incomplete cases illustrate feasibility and learning-curve considerations, although procedure times may be longer and evidence remains limited relative to conventional colonoscopy [42]. Clinical adoption will therefore depend on demonstrating not only technical feasibility but also clear advantages in patient comfort, completion rates, operator workload, workflow integration, and procedural efficiency [12, 42]. Importantly, the translational challenge differs from that of AI: whereas AI can often be layered onto an established colonoscopy workflow, robotic assistance may alter the physical interface, navigation strategy, equipment requirements, and division of tasks between operator and system [12, 15]. Physical augmentation therefore carries potentially greater workflow and implementation consequences, even when its intended objective—reducing human workload—is analogous to that of cognitive augmentation [12, 38]. The principal robotic approaches discussed in this review are compared in Table 1 according to their locomotion or actuation strategy, degree of human–system control, intended human-factor benefit, evidence maturity, and principal translational limitations.

**Table 1:** Comparison of principal robotic approaches for colonoscopy

Robotic approach	Locomotion / actuation	Human-system control	Intended advantage	Evidence maturity	Principal translational limitations	References
Self-propelled / inchworm systems	Sequential anchoring and propulsion	Operator-supervised advancement	Reduced push force and looping; potential reduction in patient discomfort and operator physical demand	Early clinical / feasibility	Procedure time, device complexity, setup and workflow integration	[12, 42]
Soft-robotic double-balloon systems	Pneumatic anchoring with sequential locomotion	Operator-controlled / assisted	Alternative to push-based advancement; potential reduction in wall stress and force transmission	Preclinical / experimental	Limited clinical validation; engineering and system-integration requirements	[43, 12]
Electromagnetically actuated soft-tethered systems	External electromagnetic actuation with visual-servo control	Semi-autonomous navigation under human supervision	Navigation assistance, lumen centring and potential reduction in operator workload	Preclinical / experimental	External actuation and sensing infrastructure; clinical effectiveness not yet established	[15]
Magnetic and capsule-based robotic concepts	Magnetic steering, external actuation, or capsule-based locomotion	Operator-controlled to assisted navigation, depending on platform	Reduced dependence on conventional push-based shaft advancement; potential comfort and ergonomic benefits	Experimental / early translational	Navigation and control reliability, therapeutic capability, external hardware and workflow integration	[12]

#### Cognitive workload in colonoscopy

*Cognitive workload* includes spatial navigation in a visually ambiguous, deformable lumen; continuous visual search for subtle lesions; dynamic decisions about looping, insufflation, washing, suction, and patient repositioning; coordination with the team; sedation-risk awareness; and rapid judgement about whether to biopsy, resect, refer, or repeat inspection [44]. Additionally, colonoscopy is a socio-technical work: outcomes emerge from interactions among people (endoscopists, nurses, technicians, anaesthesia staff), tasks (navigation, lesion detection, therapy, documentation), tools (endoscopes, processors, accessories), environment (room layout, noise, lighting), and organizational constraints (scheduling, throughput targets, reporting requirements). This lens is particularly relevant because many safety risks in endoscopy occur outside the narrow “scope-in” moment—e.g., consent, procedure indication, patient preparation, specimen handling, recovery monitoring, and communication of follow-up plans [45]. European guidance on safety checklists for gastrointestinal endoscopy, issued jointly by the European Society of Gastrointestinal Endoscopy and the European Society of Gastroenterology and Endoscopy Nurses and Associates, adapts the “sign in / time out / sign out” structure to endoscopy workflows, aiming to strengthen shared situational awareness, reduce communication failures, and normalise team-based verification steps [46]. Empirical implementation studies suggest that endoscopy safety checklists can improve team communication and teamwork, mechanisms plausibly linked to error reduction [47]. Contemporary endoscopy training frameworks increasingly recognise that competence extends beyond technical manoeuvres to include team communication, situational awareness, workflow literacy, decision-making, and other non-technical skills [48–52]. Cognitive-load research further demonstrates that procedural workload is influenced not only by task complexity but also by learner, patient, supervisor, and environmental factors, while procedure-specific instruments and eye-tracking approaches have been developed to characterise cognitive burden and visual behaviour during colonoscopy [8, 9, 53].

These principles extend to robotic surgery, where technological mediation may redistribute rather than eliminate cognitive and organizational demands [25, 41]. The physical separation of the console surgeon from the patient and bedside team can alter communication, coordination, situational awareness, and workflow [41]. Console-based operating requires sustained visual attention, coordination with the bedside team, and awareness of system state and limitations [25, 41]. Accordingly, training for technologically augmented procedures should encompass not only technical proficiency but also team communication, situational awareness, appropriate reliance on automated functions, and readiness to resume direct control when required [25, 54]. Cognitive workload should not be viewed merely as an operator limitation to be reduced by technology [25]. Rather, technological assistance redistributes cognitive demands: automation may reduce perceptual or psychomotor workload while simultaneously increasing supervisory, coordination, and system-monitoring demands [25, 38]. Successful augmentation therefore depends not simply on reducing workload, but on maintaining appropriate operator engagement and situational awareness as the nature of that workload changes [25, 54].

#### Perceptual and cognitive augmentation: AI

Among the technological domains considered in this review, AI-assisted lesion detection currently has the most mature clinical evidence base, including randomised trials, meta-analyses, and clinical guidance [13, 55, 56]. AI applications in colonoscopy can be grouped broadly into CADe for lesion detection, CADx for optical characterisation and decision support, and systems for procedural quality monitoring [11]. CADe has been evaluated extensively in randomised clinical trials and meta-analyses, consistently demonstrating improvements in adenoma detection and, in tandem-colonoscopy studies, reductions in adenoma miss rates [13, 55, 57]. However, uncertainty remains regarding the extent to which these improvements translate into long-term outcomes such as reduced post-colonoscopy colorectal cancer, as well as their implications for surveillance burden and cost-effectiveness [55, 56]. Importantly, increasing evidence also suggests that AI assistance may alter endoscopist behaviour and visual attention, raising concerns regarding cognitive offloading, overreliance, and potential deskilling [16, 58, 59].

CADx represents a distinct form of cognitive augmentation because it extends AI support from lesion detection to optical characterisation and subsequent clinical decision-making [60]. Although CADx may support strategies such as resect-and-discard or leave-in-situ for selected diminutive colorectal polyps, its clinical translation requires a higher level of confidence than detection alone because incorrect classification may directly influence treatment and surveillance decisions [60]. Evidence from systematic reviews suggests promising diagnostic performance, but performance remains dependent on lesion characteristics, image quality, system architecture, and the clinical setting, while questions regarding generalisability and responsibility for AI-supported decisions remain relevant [60]. Accordingly, CADx currently represents decision support rather than autonomous decision-making and illustrates why increasing cognitive autonomy requires progressively stronger clinical validation and human oversight [11, 60].

Quality-monitoring AI may be particularly relevant to clinician workload because it can externalise routine vigilance tasks (e.g., reminding about withdrawal inspection quality, documenting landmarks, scoring bowel preparation) and potentially standardise practice across operators [14]. For example, ENDOANGEL-derived systems have been evaluated for bowel preparation assessment and detection, while “effective withdrawal time” metrics derived from AI aim to quantify inspection quality beyond simple clock time [61]. These tools may reduce cognitive burden by handling measurement, prompting, and documentation, but they may also add visual clutter or alert fatigue if interface design ignores human cues and attentional limits [11]. These apparently opposing effects illustrate the central trade-off of cognitive augmentation [16, 58]. AI can improve measurable task performance while simultaneously altering the cognitive processes through which that performance is achieved [58, 59]. Consequently, higher detection rates alone cannot establish successful human–AI integration: evaluation should also determine whether assistance preserves independent detection capability, appropriate reliance, situational awareness, and the ability to perform effectively when AI support is unavailable or incorrect [16, 54].

#### Integrating physical, perceptual, and cognitive augmentation

Considered together, the evidence from human factors, ergonomics, AI, and robotics suggests that augmentation should not be evaluated as a collection of independent technological functions [25, 62]. These domains are interdependent: robotic assistance may reduce physical workload but modify cognitive and team demands; AI may reduce perceptual workload while introducing risks of attentional offloading and overreliance; and increasing autonomy may decrease direct task execution while increasing supervisory responsibility [25, 38, 58]. The relevant outcome is therefore not simply whether a technology improves an isolated performance metric, but how it redistributes workload, control, and responsibility across the clinician–technology system [25, 62]. This interaction also implies that benefits in one domain may be accompanied by costs in another [16, 38]. A system that improves navigation but increases monitoring burden, or improves lesion detection while weakening unaided vigilance, cannot be considered successful solely on the basis of its primary technical endpoint [38, 58]. Human–technology integration therefore requires multidimensional evaluation encompassing clinical performance, physical and cognitive workload, situational awareness, usability, team interaction, failure recovery, and preservation of human competence [22, 62]. Viewed in this way, human factors and ergonomics are not ancillary considerations in the adoption of AI and robotics, but determinants of whether technological capability translates into effective clinical augmentation [25, 62]. The maturity of evidence also differs substantially across these domains. Whereas some forms of perceptual augmentation, particularly CADe, are supported by randomized clinical evidence and meta-analyses, many forms of physical augmentation and higher-level task autonomy remain at preclinical, engineering, or early feasibility stages [12, 42, 55].

#### Proposed framework for shared autonomy in colonoscopy

**Fig. 2:**
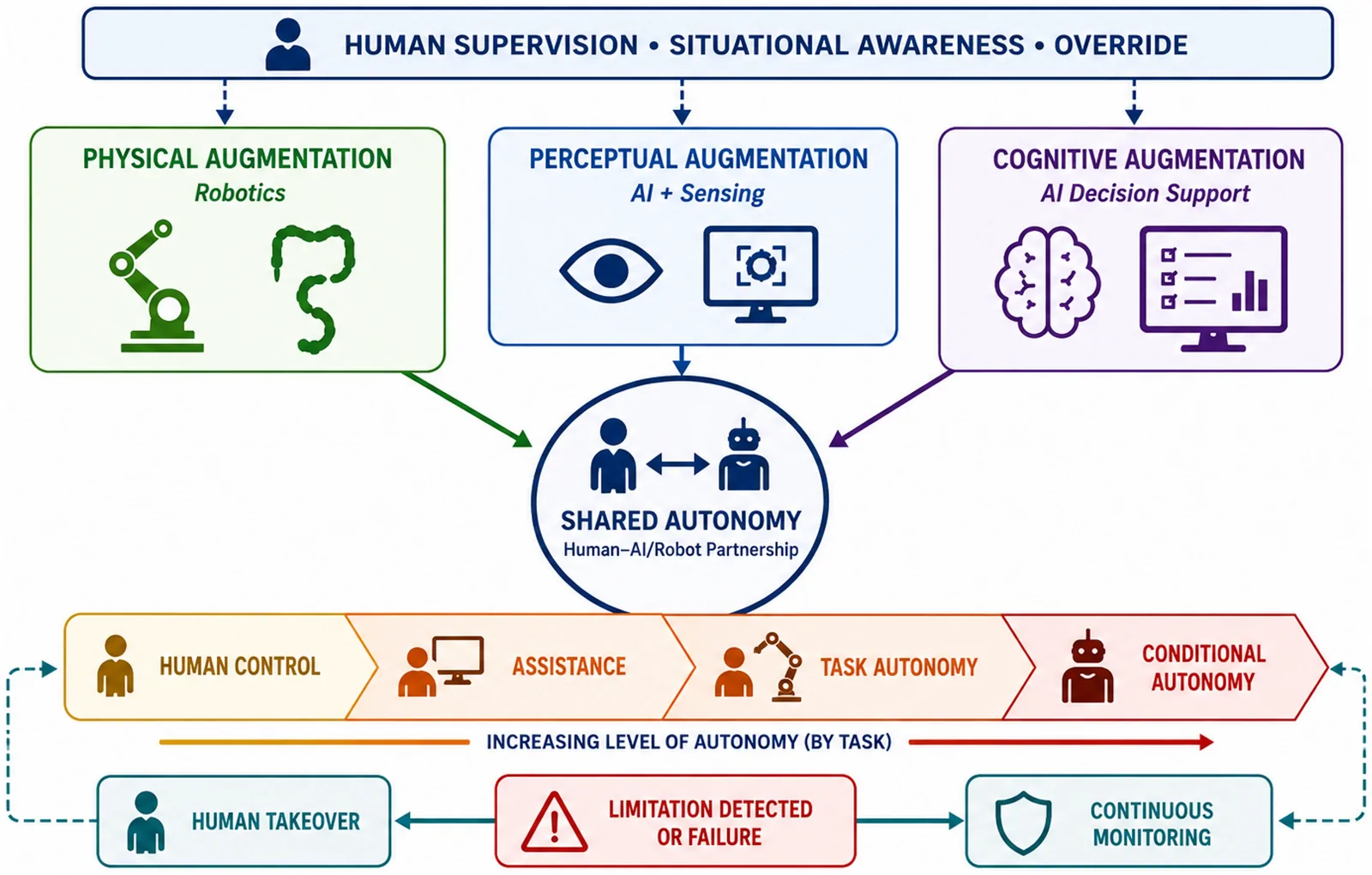
Task-based conceptual framework for *shared autonomy*. The proposed framework conceptualizes *shared autonomy* as the task-specific allocation of control between the clinician and technological systems under continuous human supervision. Three complementary forms of augmentation are represented: physical augmentation, provided primarily by robotic systems; perceptual augmentation, supported by artificial intelligence and sensing technologies; and cognitive augmentation, involving AI-based decision support. Rather than assigning a single level of autonomy to an entire procedure or platform, autonomy is considered at the task level, ranging from human control through assistance and task autonomy to conditional autonomy. Human supervision, situational awareness, and the ability to override the system remain across all levels

Building on established surgical-robotics autonomy taxonomies, we propose a task-based framework integrating three dimensions: (i) the procedural task, (ii) the type of technological augmentation involved—physical, perceptual, or cognitive—and (iii) the degree of control allocated between the clinician and the technological system. Physical augmentation addresses navigation, stability, force transmission, and operator ergonomics; perceptual augmentation supports visual inspection, lesion detection, and sensing; and cognitive augmentation supports interpretation, decision-making, quality monitoring, and procedural management. Different tasks may therefore operate at different levels of human–system control while remaining under overarching human supervision, with provision for intervention or takeover when system performance becomes inadequate (Fig. 2) [22, 23, 25].

Colonoscopy can be conceptualised as a sequence of interdependent tasks: (i) navigation and lumen progression; (ii) mucosal inspection; (iii) lesion detection; (iv) lesion characterisation; (v) therapeutic intervention; and (vi) documentation and reporting. These stages differ in their cognitive, perceptual, and psychomotor requirements and therefore in their suitability for technological assistance [22, 23]. Lower-level, repetitive, or physically demanding tasks—such as lumen centring, loop reduction, or scope advancement—represent suitable targets for robotic or soft-robotic assistance [12, 15, 43]. Similarly, perceptual tasks such as lesion detection can be augmented through AI-based systems (e.g., CADe), while quality-monitoring algorithms may support standardisation of inspection and documentation processes [13, 14]. In contrast, higher-order clinical decisions—including therapeutic strategy, risk assessment, and complication management—remain fundamentally human-driven [20, 22].

Within the proposed framework, *shared autonomy* may ultimately evolve from relatively fixed task-specific assistance towards more adaptive forms of human–system interaction [17, 63]. However, real-time workload sensing, context-aware modulation of assistance, and dynamic redistribution of autonomy remain largely conceptual or experimental and require prospective validation before their clinical value can be established [63]. From a translational perspective, this framework highlights the need to move beyond isolated performance metrics (e.g., adenoma detection rate) towards multidimensional evaluation models incorporating cognitive load, ergonomics, workflow integration, team interaction, and patient-centred outcomes [25, 62]. Such an approach aligns with human-centred design principles and provides a roadmap for the safe and effective integration of AI and robotic technologies into clinical endoscopy [62]. This task-based architecture is not unique to colonoscopy [22, 23]. A similar decomposition can be applied to multi-port and single-port robotic surgery, where camera control, instrument positioning, suturing assistance, target tracking, or selected repetitive manoeuvres may be partially automated while strategic decisions, tissue interpretation, oncological judgement, and complication management remain surgeon-led [22, 24, 25]. Importantly, *shared autonomy* in endoscopy differs from that in robotic surgery in both the nature of the tasks delegated and the procedural environment [12, 25]. Colonoscopy combines deformable intraluminal navigation with continuous visual diagnosis and therefore integrates physical assistance with perceptual and cognitive augmentation, whereas robotic surgery predominantly involves robot-mediated execution of physical tasks within a defined operative field [12, 15, 25]. Table 2 illustrates that different task-level autonomy states may coexist within the same procedure. CADe, for example, represents assistive autonomy in lesion detection, whereas robotic navigation may progress toward supervised task autonomy for predefined functions such as lumen centring or controlled advancement [13, 23, 25].

**Table 2:** Suggested task-based mapping of *shared autonomy* in colonoscopy onto the Levels of Autonomy in Surgical Robotics (LASR) framework. Taxonomy refers to the classification of Lee et al. [23]. Because the LASR taxonomy was originally developed for embodied surgical robotic systems, its application to AI-based functions such as CADe and CADx should be interpreted as an analogous task-level mapping of human–system control rather than as a literal classification of these technologies as surgical robots

Colonoscopy task	Current technology	Human role	Technology role	Approximate LASR level	Evidence maturity
Navigation/advancement	Robotic/self-propelled colonoscopes	Supervises progression, safety, intervention	Propulsion/navigation assistance	1–2	Experimental/early clinical
Lumen centring/stabilisation	Visual servoing/robotic control	Supervises and overrides	Executes defined navigation subtask	2	Experimental
Mucosal inspection	AI quality monitoring	Performs inspection and interprets context	Monitors exposure/withdrawal quality	1–2	Clinical
Lesion detection	CADe	Confirms and evaluates detected target	Detects/highlights suspected lesion	1	High clinical
Lesion characterisation	CADx	Integrates output and makes clinical decision	Provides optical prediction	1	Clinical/emerging
Therapeutic intervention	Conventional/robot-assisted tools	Selects strategy and executes/controls treatment	Stabilisation or task assistance	1–2	Early/variable
Documentation	AI-assisted reporting	Reviews and validates	Generates measurements/report elements	1–2	Emerging

#### Human–AI–robot interaction: failure, oversight, and recovery

U.S. regulatory documentation for AI polyp detection systems explicitly states that such devices are designed to aid lesion detection in real time and are not intended to replace clinical decision making (FDA DEN200055; FDA K211951). This distinction is not semantic; it defines responsibility allocation, training needs, and evaluation endpoints. Augmentative tools must handle substantial real-time information flow (continuous video, alerts, quality metrics, documentation prompts) without overwhelming attention or degrading team communication [16, 46]. Human-centred evaluation frameworks in clinical decision support stress usability, interpretability, workflow fit, and safety monitoring—not merely algorithmic accuracy—because poorly integrated AI can increase cognitive load and create new error pathways [64]. From a preventive-care perspective, augmentation also extends beyond the procedure: integrating AI outputs into structured reporting, audit-and-feedback systems, and screening program pathways may improve both intervention quality and population prevention effectiveness, but only if data governance, interoperability, and implementation constraints are addressed [11].

A critical consideration in human–AI–robot interaction is failure management [54, 62]. Task allocation creates new failure modes because errors may originate from the technological system, the human operator, or their interaction [54, 62]. AI may fail through false-positive or false-negative detection, incorrect characterisation, or inappropriate reliance on its output, whereas robotic assistance may fail through loss of sensing, actuation, navigation, or system availability [16, 62]. Human failure may conversely involve failure to recognise an incorrect system output, delayed intervention, inappropriate override, or overreliance on automation [25, 54]. Such systems should therefore incorporate explicit failure-recovery pathways: technological assistance should remain transparent to the operator, loss or uncertainty of automated function should be identifiable, and control should be recoverable by the clinician whenever technically feasible [54, 62]. Importantly, human oversight should not be interpreted as a sufficient safeguard by itself, because effective rescue requires maintained operator competence, situational awareness, and the ability to recognise when technological assistance has failed [62].

#### Clinical implications of shared autonomy: training, responsibility, and workflow

Increasing task autonomy has implications extending beyond technical performance [20, 25]. First, training paradigms will need to evolve from acquisition of procedural skills alone toward competency in human–robot collaboration [25, 54]. Clinicians will need to understand not only how to operate assistive systems, but also their operational boundaries, failure modes, appropriate levels of reliance, and conditions requiring human takeover [54, 62]. This requirement becomes increasingly important as the clinician transitions from direct task execution toward supervisory control, because preservation of sufficient procedural competence remains necessary to intervene when automated assistance becomes unreliable or fails [25]. Second, progressive autonomy challenges traditional models of professional responsibility [20]. In conventional colonoscopy and teleoperated robotic surgery, responsibility remains predominantly attributable to the clinician controlling the procedure [20]. As systems acquire greater capacity to select or execute specific actions, however, responsibility may become distributed across clinicians, institutions, manufacturers, and software developers [20]. Existing analyses of surgical robotics have therefore identified increasing autonomy as a challenge for regulatory classification, accountability, and liability [20]. A task-based model may provide greater conceptual clarity by defining, for each component of the procedure, which agent selects the action, which agent executes it, who monitors performance, and under what circumstances control must return to the clinician [20, 23]. Finally, *shared autonomy* is likely to modify procedural workflow and team organisation [41]. Robotic operating rooms already require redistribution of roles between console surgeons, bedside assistants, nursing personnel, and technical support [41]. Increasing automation introduces an additional supervisory function: the clinical team must understand when the system is acting autonomously, how its actions are communicated, and how control can be interrupted or transferred [25, 41]. Similar principles apply to colonoscopy, where simultaneous AI-based detection, quality monitoring, robotic navigation, and automated documentation could potentially reduce selected components of cognitive and physical workload but could also generate new demands related to interface management, alert prioritisation, and coordination [16, 25]. These systems should therefore be evaluated not only according to task performance, but also according to their effects on training, situational awareness, team communication, workflow, and responsibility allocation [25, 41, 62] Table 3.

**Table 3:** Potential pitfalls of *shared autonomy* according to the different tasks in colonoscopy

Task	Technology	Human role	Technology role	Autonomy	Failure	Fallback
Navigation	Robotic propulsion	Supervision	Advancement	Task autonomy	Tracking/propulsion failure	Manual takeover
Mucosal inspection	AI quality monitoring	Performs inspection and interprets context	Monitors exposure/withdrawal quality	Assistive	False reassurance or failure to identify incomplete exposure	Independent human inspection / repeat inspection
Lesion detection	CADe	Confirmation	Detection	Assistive	False negative/positive	Independent human inspection
CADx	optical diagnosis	Final decision	Classification	Assistive	Misclassification	Clinician interpretation
Therapeutic intervention	Conventional/robot-assisted tools	Selects strategy and performs/supervises treatment	Stabilisation or constrained task assistance	Human-controlled / assistive	Incorrect guidance, instrument or actuation failure	Immediate human override / conventional completion
Documentation	AI reporting	Validation	Automated capture	Task autonomy	Incorrect data	Clinician correction

## Conclusions

Future colonoscopy systems should not be framed as replacements for clinicians, but as human-centred technologies that support clinicians across selected cognitive and physical procedural tasks [23, 25]. Within a *shared-autonomy* framework, AI and robotic systems may support lesion detection, optical characterization, quality monitoring, navigation, stability, and force control, while the clinician retains responsibility for clinical interpretation, therapeutic strategy, patient safety, and management of complications. Evidence supports CADe-associated improvements in detection metrics and emerging quality-monitoring capabilities; however, real-world deployment also raises risks of alert fatigue, overreliance, and potential deskilling, requiring careful training, governance, and continuous performance monitoring [56]. Robotic and soft-robotic colonoscopy may reduce forceful manipulation, operator strain, and patient discomfort, and may ultimately support assisted or semi-autonomous navigation. Nevertheless, clinical evidence remains immature, and these technologies must be evaluated against conventional colonoscopy using multidimensional outcomes, including diagnostic performance, procedural quality, patient-centered outcomes, workflow integration, cost, maintenance, usability, and clinician wellbeing [12].

## Supplementary Information

Below is the link to the electronic supplementary material.

Supplementary file1 (DOCX 24 kb)

## Data Availability

No datasets were generated or analysed during the current study.
