# Supplementary material for: Augmenting colonoscopy with artificial intelligence and robotic assistance: a human-factors scoping review toward a task-based shared autonomy paradigm
Source: J Robot Surg. 2026 Oct 12;20(1):1090. doi: 10.1007/s11701-026-03923-3 (PMC13654901; doi:10.1007/s11701-026-03923-3)
Supplement: Supplementary file 1 [file 11701_2026_3923_MOESM1_ESM.docx]

**Supplementary Table 1. Full electronic search strategies**

| **Database** | **Platform** | **Coverage** | **Search date** | **Complete search strategy** | **Results retrieved** |
| --- | --- | --- | --- | --- | --- |
| **PubMed** | **National Library of Medicine** | Database inception  to  August 2026 | 15/08/2026 | ((colonoscop*[tiab] OR "gastrointestinal endoscop*"[tiab] OR "GI endoscop*"[tiab]) AND ("human factor*"[tiab] OR "cognitive load"[tiab] OR "cognitive workload"[tiab] OR "mental workload"[tiab] OR "non-technical skill*"[tiab] OR teamwork[tiab] OR "situational awareness"[tiab] OR ergonom*[tiab] OR musculoskeletal[tiab] OR "physical workload"[tiab] OR "artificial intelligence"[tiab] OR "machine learning"[tiab] OR "deep learning"[tiab] OR "computer-aided detection"[tiab] OR "computer aided detection"[tiab] OR CADe[tiab] OR "computer-aided diagnosis"[tiab] OR "computer aided diagnosis"[tiab] OR CADx[tiab] OR "quality monitoring"[tiab] OR robot*[tiab] OR "robotic colonoscopy"[tiab] OR "robotic endoscopy"[tiab] OR "soft robot*"[tiab] OR "self-propelled"[tiab] OR autonom*[tiab] OR "semi-autonomous"[tiab] OR "shared autonomy"[tiab] OR "shared control"[tiab] OR "human-robot interaction"[tiab] OR "human robot interaction"[tiab] OR "human-robot collaboration"[tiab] OR "human robot collaboration"[tiab])) OR (("surgical robot*"[tiab] OR "robotic surg*"[tiab] OR "robot-assisted surg*"[tiab] OR "robot assisted surg*"[tiab]) AND (autonom*[tiab] OR "level* of autonomy"[tiab] OR "task autonomy"[tiab] OR "shared autonomy"[tiab] OR "shared control"[tiab] OR "human-robot interaction"[tiab] OR "human robot interaction"[tiab] OR "human-robot collaboration"[tiab] OR "human robot collaboration"[tiab] OR teleoperat*[tiab] OR "supervisory control"[tiab])) | 3071 |
| **Embase** | **Elsevier Embase.com** | Database inception  to  August 2026 | 15/08/2026 | ((('colonoscopy'/exp OR 'gastrointestinal endoscopy'/exp OR colonoscop*:ti,ab,kw OR 'gastrointestinal endoscop*':ti,ab,kw OR 'GI endoscop*':ti,ab,kw) AND ('human factor*':ti,ab,kw OR 'cognitive load':ti,ab,kw OR 'cognitive workload':ti,ab,kw OR 'mental workload':ti,ab,kw OR 'non-technical skill*':ti,ab,kw OR teamwork:ti,ab,kw OR 'situational awareness':ti,ab,kw OR ergonom*:ti,ab,kw OR musculoskeletal:ti,ab,kw OR 'physical workload':ti,ab,kw OR 'artificial intelligence':ti,ab,kw OR 'machine learning':ti,ab,kw OR 'deep learning':ti,ab,kw OR 'computer-aided detection':ti,ab,kw OR 'computer aided detection':ti,ab,kw OR CADe:ti,ab,kw OR 'computer-aided diagnosis':ti,ab,kw OR 'computer aided diagnosis':ti,ab,kw OR CADx:ti,ab,kw OR 'quality monitoring':ti,ab,kw OR robot*:ti,ab,kw OR 'robotic colonoscopy':ti,ab,kw OR 'robotic endoscopy':ti,ab,kw OR 'soft robot*':ti,ab,kw OR 'self-propelled':ti,ab,kw OR autonom*:ti,ab,kw OR 'semi-autonomous':ti,ab,kw OR 'shared autonomy':ti,ab,kw OR 'shared control':ti,ab,kw OR 'human-robot interaction':ti,ab,kw OR 'human robot interaction':ti,ab,kw OR 'human-robot collaboration':ti,ab,kw OR 'human robot collaboration':ti,ab,kw)) OR (('surgical robot*':ti,ab,kw OR 'robotic surg*':ti,ab,kw OR 'robot-assisted surg*':ti,ab,kw OR 'robot assisted surg*':ti,ab,kw) AND (autonom*:ti,ab,kw OR 'level* of autonomy':ti,ab,kw OR 'task autonomy':ti,ab,kw OR 'shared autonomy':ti,ab,kw OR 'shared control':ti,ab,kw OR 'human-robot interaction':ti,ab,kw OR 'human robot interaction':ti,ab,kw OR 'human-robot collaboration':ti,ab,kw OR 'human robot collaboration':ti,ab,kw OR teleoperat*:ti,ab,kw OR 'supervisory control':ti,ab,kw))) | 10303 |
| **Scopus** | **Elsevier** | Database inception  to  August 2026 | 15/08/2026 | (TITLE-ABS-KEY(colonoscop* OR "gastrointestinal endoscop*" OR "GI endoscop*") AND TITLE-ABS-KEY("human factor*" OR "cognitive load" OR "cognitive workload" OR "mental workload" OR "non-technical skill*" OR teamwork OR "situational awareness" OR ergonom* OR musculoskeletal OR "physical workload" OR "artificial intelligence" OR "machine learning" OR "deep learning" OR "computer-aided detection" OR "computer aided detection" OR CADe OR "computer-aided diagnosis" OR "computer aided diagnosis" OR CADx OR "quality monitoring" OR robot* OR "robotic colonoscopy" OR "robotic endoscopy" OR "soft robot*" OR "self-propelled" OR autonom* OR "semi-autonomous" OR "shared autonomy" OR "shared control" OR "human-robot interaction" OR "human robot interaction" OR "human-robot collaboration" OR "human robot collaboration")) OR (TITLE-ABS-KEY("surgical robot*" OR "robotic surg*" OR "robot-assisted surg*" OR "robot assisted surg*") AND TITLE-ABS-KEY(autonom* OR "level* of autonomy" OR "task autonomy" OR "shared autonomy" OR "shared control" OR "human-robot interaction" OR "human robot interaction" OR "human-robot collaboration" OR "human robot collaboration" OR teleoperat* OR "supervisory control")) | 10067 |

**Abbreviations:** tiab, title and abstract; ti, title; ab, abstract; kw, keyword; TITLE-ABS-KEY, title, abstract, and keywords; CADe, computer-aided detection; CADx, computer-aided diagnosis.

**Notes**: Search strategies were adapted to the syntax and indexing system of each database. The reported numbers represent records retrieved before deduplication. Searches were conducted from database inception to 15 August 2026. No publication-date restrictions were applied.
